# Supplementary material for: Coherent quantum control of nitrogen-vacancy center spins near 1000 kelvin
Source: Nat Commun. 2019 Mar 22;10:1344. doi: 10.1038/s41467-019-09327-2 (PMC6430790; doi:10.1038/s41467-019-09327-2)
Supplement: Supplementary file 1 — Supplementary information [file 41467_2019_9327_MOESM1_ESM.pdf]

Supplementary Information

for

Coherent quantum control of nitrogen-vacancy center spins near 1000 kelvin

Liu et al.

Supplementary Note 1 | Nanodiamond and Ni nanoparticle.

Nanodiamonds (NDs) with ensemble nitrogen-vacancy (NV) centers were purchased from Adámas, with initial concentration of 1 mg ml^{-1} (water solution). The average size of NDs is about 140 nm from dynamic light scattering (DLS) measurement. Each ND contains about 500 NV centers. To prepare the sample for high-temperature ODMR (HiT ODMR), a drop of 10 μl ND ethanol solution (5 μg ml^{-1}) was transferred to a transmission electron microscopy (TEM) grid (Ted Pella) with pipette. NDs attached to the amorphous carbon film by Van der Waals' force after the ethanol volatilized (in several minutes, see Fig. 1A inset of the main text for a typical TEM image of a bare ND on the carbon film). ND appears to be stable on the carbon film, with no change observed during the whole measurement.

Nickle nanoparticles (NPs) were obtained by ball milling of Ni powder (3-7 μm, Strem Chemicals). High temperature annealing (973 K, 2 hours) was performed in 10% H_2/Ar after ball milling to improve the crystallinity of the NPs. After size sorting by centrifugation, Ni NPs with size of about 100 nm were dispersed in ethanol and dropped on a TEM grid with the same method as for depositing NDs on a TEM grid. Supplementary Figure 1 shows a typical TEM image of a single Ni NP and its energy-dispersive X-ray spectroscopy (EDX) data.

The locations of Ni NPs and NDs in the proximity were identified by TEM imaging, as shown in Supplementary Figure 2 below and Fig. 5B of the main text. On a TEM grid, the amorphous carbon film forms unique net patterns (Supplementary Figure 2A), which serves as the natural markers to locate the nanoparticles in the confocal image. The confocal image (Supplementary Figure 2B) shows that the NDs are much brighter than the carbon film and the Ni NPs. The overlap between the TEM and confocal images (Supplementary Figure 2C) was employed to locate NDs with Ni NPs in the proximity (Supplementary Figure 2D).

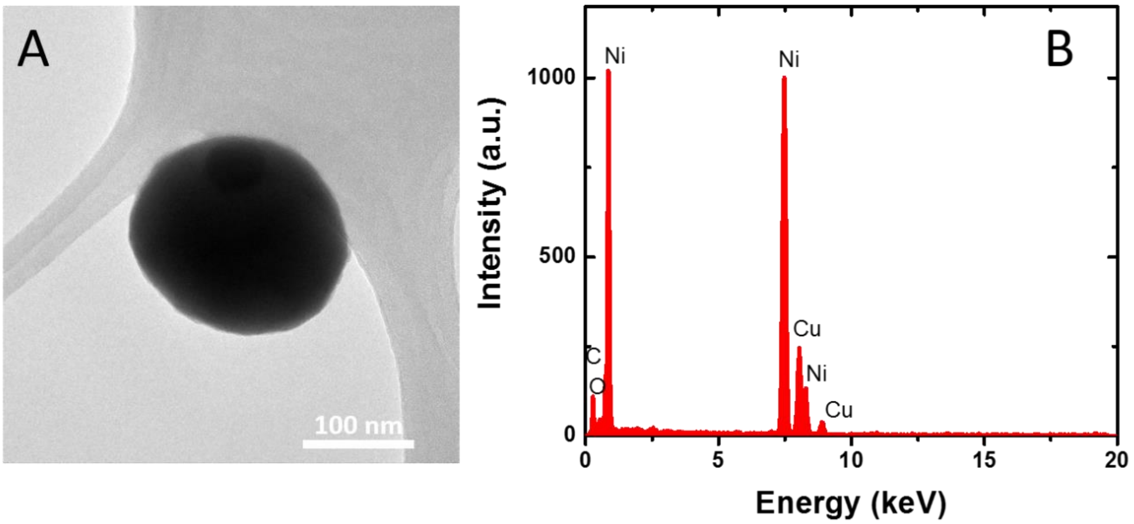

Supplementary Figure 1 | A typical Ni nanoparticle on a carbon film. (**A**) The TEM image of a Ni NP (black sphere) on a carbon film (gray frame). (**B**) The EDX spectrum shows that the NP is pure Ni, with Cu and carbon signals coming from the TEM grid.

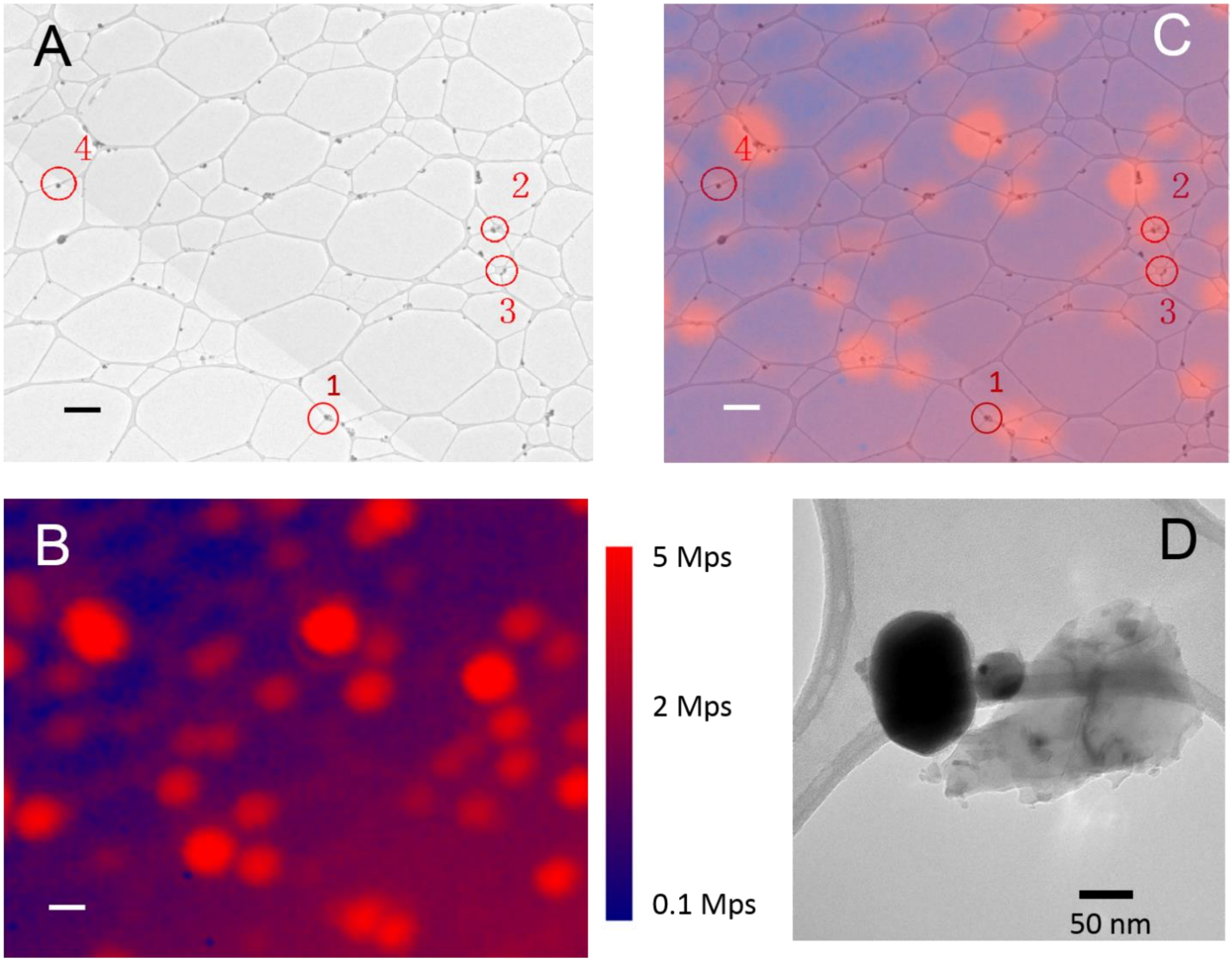

Supplementary Figure 2 | Identifications of NDs and Ni NPs by overlap of TEM and confocal images. (**A**) The TEM image of the NDs and Ni NPs on a carbon film. The gray mesh pattern is the amorphous carbon film. The darker dots on the carbon film are NDs or Ni NPs (**B**) The confocal image of NDs and Ni NPs on a TEM grid (the same region as in A). Color bar shows the fluorescence counts of each pixel. The bright spots are NDs with ensemble NV centers. The carbon film and the Ni NPs have much weaker fluorescence than the NDs. **(C)** Overlap of the TEM and confocal images. In A, B and C, four Ni NPs with nearby NDs are circled and labelled. The scale bars in A, B and C are 1 μm. (**D**) Close-up TEM image of an ND and Ni NPs (No. 3 in **A**). The Ni NPs (sphere-shaped) have darker contrast than the ND (triangle-shaped).

Supplementary Note 2 | Sample chamber

To avoid oxidation of the amorphous carbon film during laser heating, the sample was protected in an argon (Ar) atmosphere in the high-temperature measurements. The chamber was built on a confocal dish, with a reusable cover, as shown in Supplementary Figure 3. The bottom of the dish was removed, then it was glued to the PCB board with microwave transmission lines. A 25-μm-diameter copper wire soldered to the transition line buried in between the dish and the PCB

board, which was employed to delivery microwave to the sample. The bottom side of the dish was finally closed with a cover glass, which formed the optical window for microscopy. The TEM grid was fixed in the chamber, and then the opened chamber was placed in glovebox of an Ar atmosphere for about 10 hours to be filled with Ar. Finally, the chamber was closed with the dish cover and sealed with additional glue before being taken out from the glovebox for ODMR measurement.

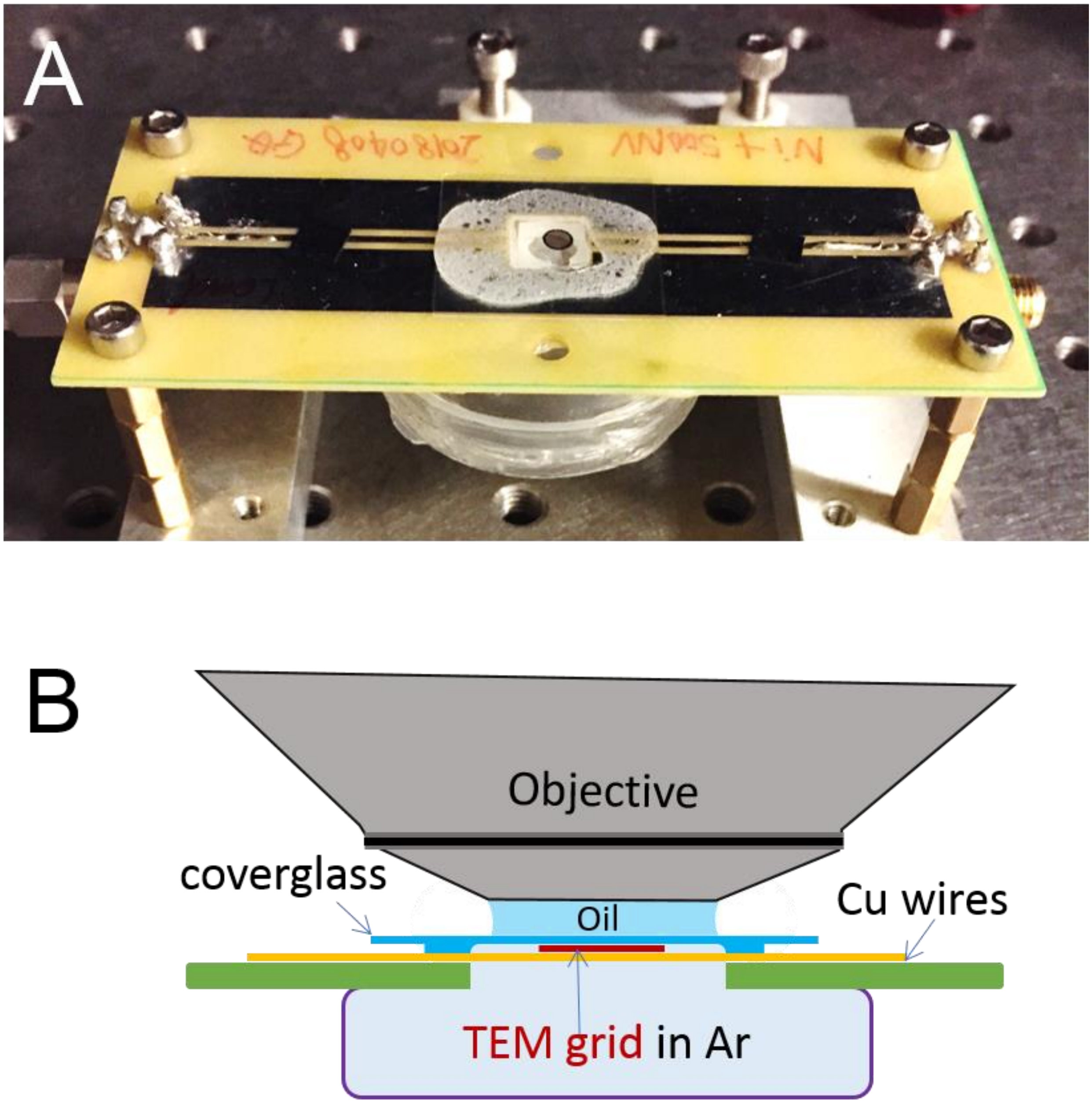

Supplementary Figure 3 | A home-built sample chamber mounted on a PCB board. (A) Photograph of the sample chamber. **(B)** Diagram of the sample chamber in ODMR measurement.

Supplementary Note 3 | ODMR setup with NIR laser heating

As shown in Supplementary Figure 4A, the optical system contained 3 parts: (1) spin polarization with a green laser (532 nm), (2) spin readout with fluorescence collection under 532 nm laser excitation, and (3) local and instantaneous heating with an NIR laser (808 nm). The 532-nm laser was modulated by an acousto-optic modulator (AOM) and coupled into a microscopy frame through a single-mode fiber. A pair of galvo mirrors were used to control the focusing position (X-Y) of the green laser, and a piezo stage was used to control the focus depth (Z) of the oil objective (NA=1.35). The fluorescence of NV centers in NDs was collected by the same objective and passed through two dichroic mirrors (DM, one for the green laser and another for the NIR laser) and filters. The fluorescent signals were converted into digital signals by a single photon counting module (SPCM, Excelitas) and then recorded by a digital counter (USB-6211, National Instruments). The NIR laser was independently controlled with an AOM and a pair of galvo mirrors, adjusted to overlap with the green laser at the DM2 position. A pair of moving lenses (orange dash box) were used to compensate the chromatic aberration between the two lasers.

Microwave (MW) pulses synchronized with the optical pulses were used to manipulate the spin states of the NV centers. The amplitude and frequency of the MW signal were controlled by the signal generator (N5181A, Agilent), and the shape of the MW pulses was modulated by an RF switch (ZASW-2-50DR, Mini Circuits). After amplification, the MW pulses were delivered to the sample through a coaxial cable, transmission lines on the sample holder, and a 25-μm-diameter copper wire. The laser excitation, NIR heating, MW manipulation, and fluorescence readout were synchronized with TTL signals from a pulse generator (PulseBlasterESR-PRO, SpinCore).

Typical zero-field continuous-wave (CW) ODMR spectra of a bare ND with ensemble NV centers are presented in Supplementary Figure 4B. The splitting of the peak is caused by the local strain of the diamond lattice. Local temperature was tuned by applying an NIR laser of different power. The shift of the resonant frequencies was induced by NIR laser heating. The temperature depends approximately linearly on the NIR laser power in the measured range (Supplementary Figure 4C). When temperature of the ND was close to 550 K ($D < 2840$ MHz), the contrast of the ODMR spectrum begun to decrease (Supplementary Figure 4C), similar to observation in Supplementary Reference (*1*).

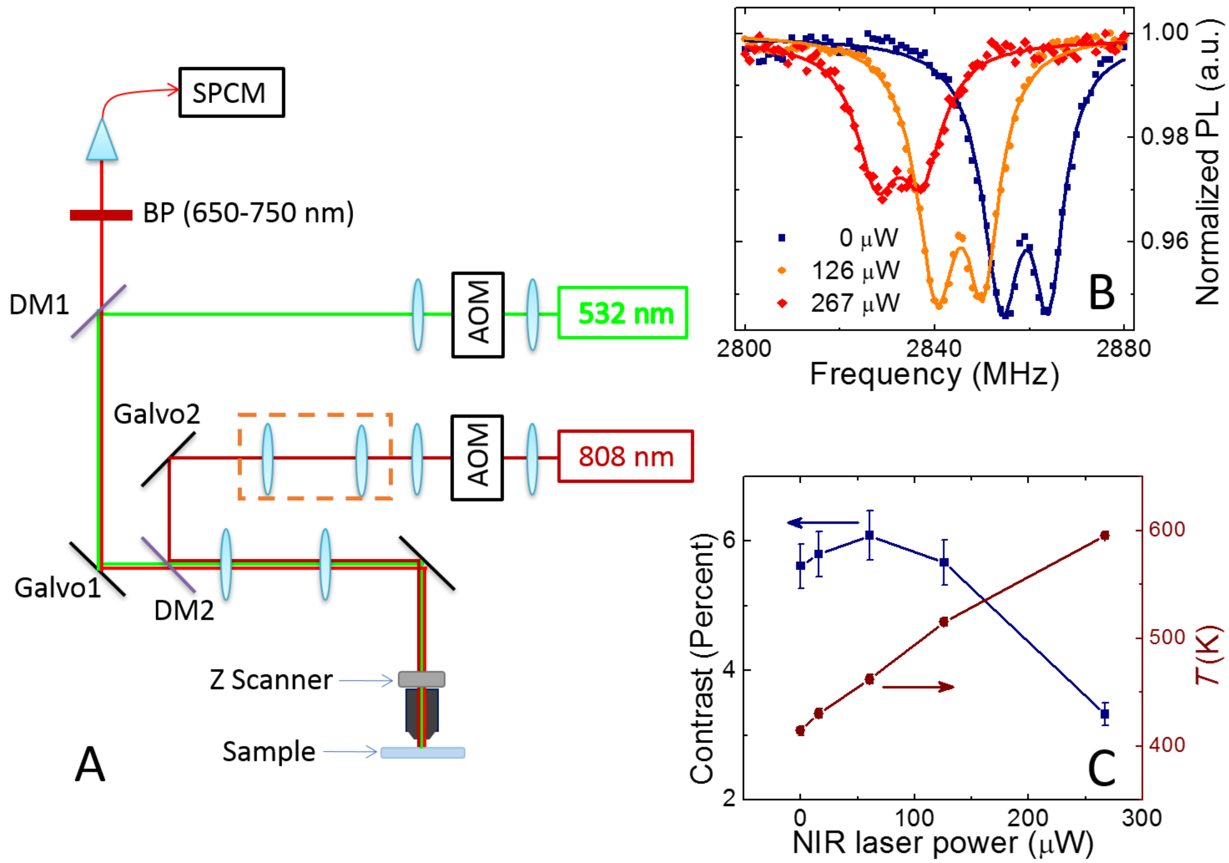

Supplementary Figure 4 | ODMR setup and typical ODMR spectra. (**A**) Diagram of the confocal system. AOM: acousto-optic modulator. DM: dichroic mirrors. BP: bandpass filter. Galvo: Scanning Galvo Mirrors. SPCM: single photon counting module. (**B**) Typical zero-field ODMR spectra of ensemble NV centers in an ND with NIR laser heating of different powers. (**C**) The temperature and contrast of ODMR spectra as functions of the NIR laser power. Error bars corresponding to the standard fitting errors.

Supplementary Note 4 | NIR laser heating

The NIR laser heating was first characterized by heating imaging. As shown in Fig. 1B of the main text and Supplementary Figure 5A, the three-point ODMR (*2*) was measured as the NIR laser beam was scanned around a selected ND. The three microwave frequencies were chosen as: f_1 and f_2 at the half-maximum points of the spin resonance with the NIR laser turned off and f_3 far away from the resonance frequency. The photon counts for the three microwave frequencies $f_{1/2/3}$ are denoted as $c_{1/2/3}$. The relative contrast change $C = (c_1 - c_2)/(2c_3 - c_1 - c_2)$ is an approximatively linear function of the resonance frequency shift if it is not too large (e.g., within in the ODMR width). Thus, the C value was converted to temperature, with calibration by two full ODMR spectra: one measured with the NIR laser turned off and one measured at the highest temperature. The patterns in the heat mapping image (Fig. 1B of main text and Supplementary

Figure 5A) correspond to the structures of the amorphous carbon films. With this method, we optimized the position of the NIR laser beam for heating efficiency. Usually the optimal position overlapped with the selected ND (e.g., the black box in Supplementary Figure 5A).

We studied the NIR heating dynamics by heat conduction measurement. Supplementary Figure 5B shows the temperature of an ND (measured with the same sequence as in Fig. 1C of the main text) as a function of the duration of the heating NIR pulse for various NIR laser focus spots on the amorphous carbon film. For each focus spot and each heating duration, a full ODMR spectrum was measured and the zero-field splitting (ZFS) *D* was extracted by Lorentzian fitting. When the NIR laser was away from the ND position (P_0), it took a delay time after the heating pulse for the temperature of the ND to rise, and the stationary temperature was lower (i.e., the final state *D* was larger). The delay time, which increases linearly with the distance (Supplementary Figure 5C), is attributed to the propagation of heat from the heating spot to the ND through the amorphous carbon film. Note that there was an extra delay in the experimental data (shadowed regions of Supplementary Figure 5B-C), which was induced by the NIR AOM delay of our setup.

All the *D* data are well fit with an exponential decay with the decay time nearly the same in the measured temperature range (300 – 380 K). In this temperature range, the ZFS *D* is an approximately linear function of the temperature. The exponential decay can be well understood with a rate equation. We define the following parameters:

T_0 - initial temperature of the ND

T_E - temperature of the environment

W - heating rate, determined by NIR laser power

γ - cooling rate, determined by the thermal conductivity of the amorphous carbon film.

The heating/cooling dynamics is determined by the rate equation

$$\frac{dT}{dt} = W - \gamma(T - T_E). \quad (1)$$

Thus, the temperature of the ND is

$$T = T_E + \frac{W}{\gamma} + \left(T_0 - T_E - \frac{W}{\gamma}\right)\exp(-\gamma t). \quad (2)$$

The local temperature increases (decreases) exponentially as the NIR laser is turned on (off), with a time scale γ^{-1}, before it reaches the stationary value ($T_E + W/\gamma$).

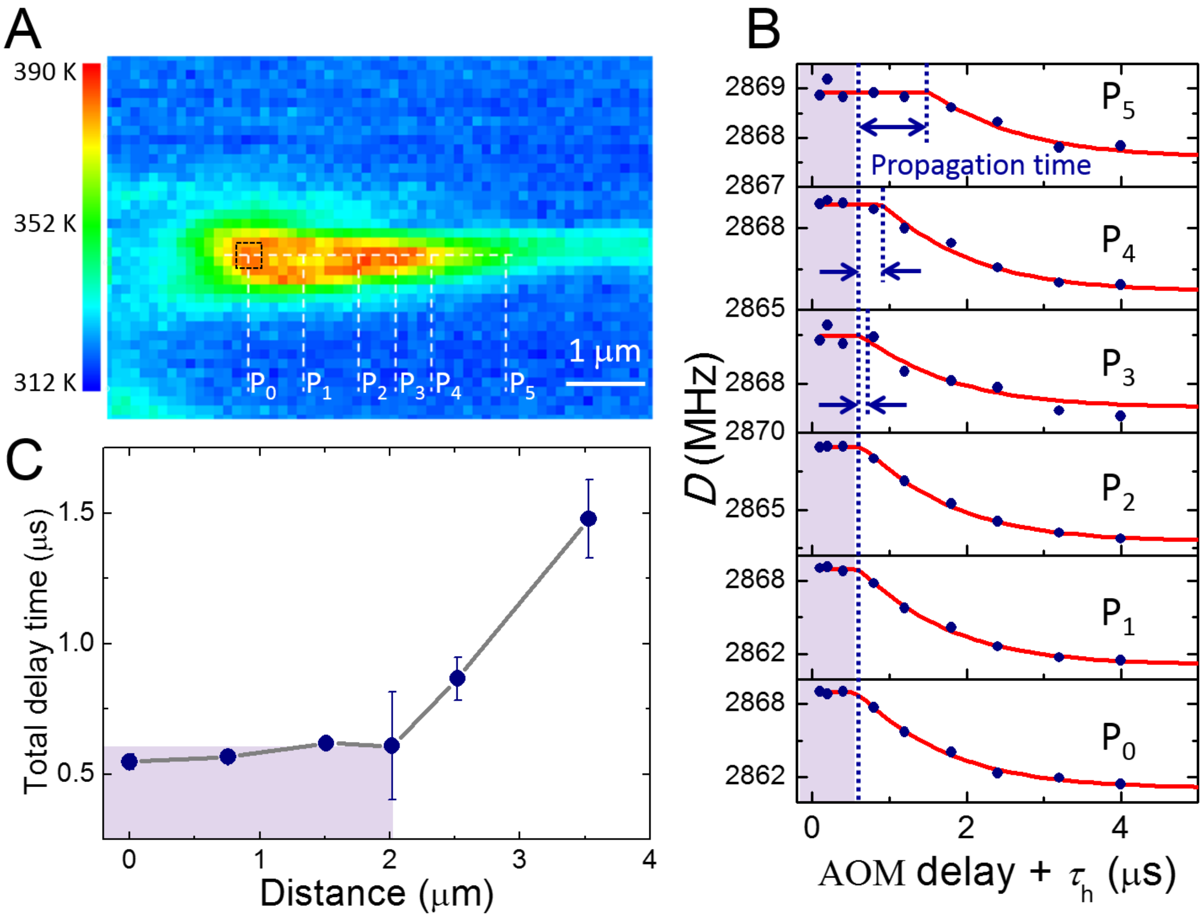

Supplementary Figure 5 | Mechanism of NIR heating. **(A)** Two-dimensional heating image. Temperature measured by an ND (indicated by the black box) as a function of the position of the NIR laser beam. To speed up the imaging process, three-point CW ODMR was used. **(B)** Zero-field splitting *D*, extracted from full ODMR spectra, as a function of the NIR heating pulse duration for 5 different NIR heating positions (marked in **A**). Pulse sequence in Fig. 1C of the main text was used, with waiting time between the heating pulse and microwave pulse t_w= 0 μs and cooling time t_c=2 μs before the readout. The shadowed region indicates the AOM delay. **(C)** The delay time for the ND temperature to rise after the NIR pulse (deduced from the fitting curve in **B**) as a function of the distance between the ND and the NIR laser focus spot. The 500-ns minimum value was due to the AOM delay. Error bars corresponding to the standard fitting errors.

Supplementary Note 5 | Mechanism of HiT ODMR

Supplementary Figure 6 presents the ODMR spectra of an ND with the temperatures independently set during spin polarization and readout. The pulse sequences are presented in Supplementary Figure 6A. For the NIR power used, the stationary temperature was above 700 K (D < 2815 MHz). At such a high temperature, both the fluorescence of NV centers and the

contrast were suppressed, and there was no ODMR signal when both the polarization and readout were carried out at high temperature (Supplementary Figure 6B, line 1). The fluorescence signal could be recovered when the readout was carried out at $T < 550$ K, but there was still no ODMR signal if the polarization pulse was applied at high temperature (line 3). On the contrary, the ODMR signal was observed at 2806 MHz when the polarization was carried out at low temperature even when the spin was read out at high temperature (line 2). These results indicate that HiT ODMR in the ND was mainly hindered by the inefficient spin polarization at high temperature. More importantly, the observed ODMR signal indicates that the spin polarization was well preserved during the fast heating and cooling processes. Optimal photon counts and ODMR contrast were achieved by performing both polarization and readout at low temperature (Supplementary Figure 6B, line 4). Thus, such a sequence (Fig. 2A) was used in the experiments shown in the main text.

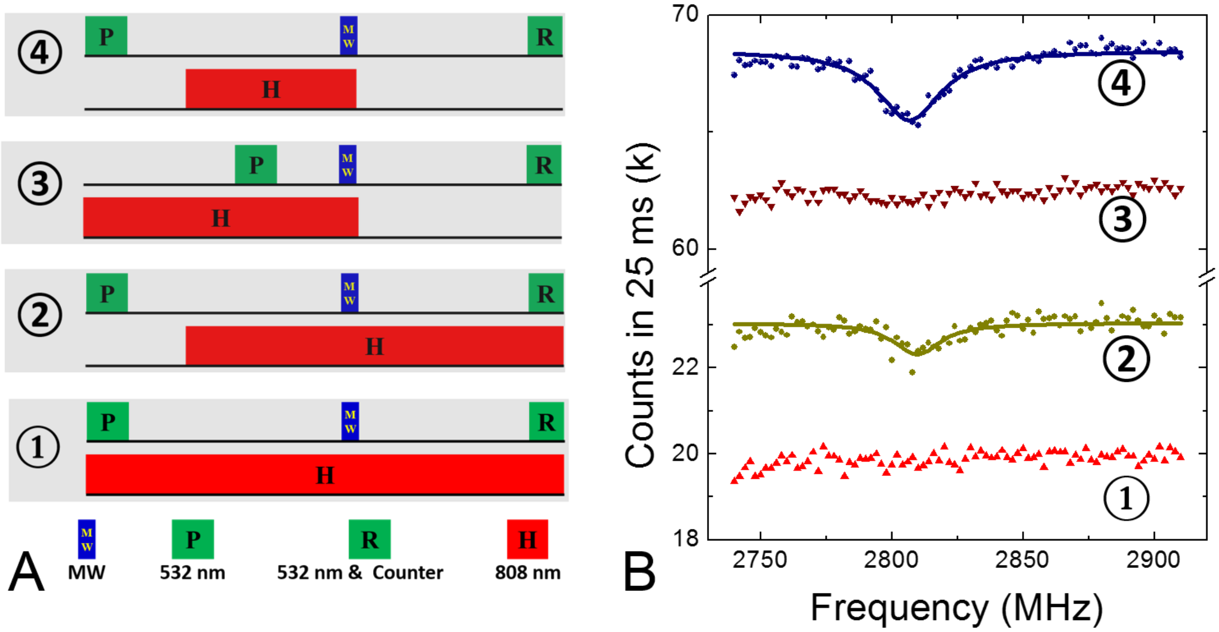

Supplementary Figure 6 | Mechanism of HiT ODMR. (**A**) Pulse sequences. The NV center spins were polarized (P) and read out (R) by the green laser and manipulated with the microwave pulse (MW). The ND was heated by an NIR pulse (H) with tunable duration and delay time. Sequence 1: polarization at high *T*, readout at high *T*; Sequence 2: polarization at low *T*, readout at high *T*; Sequence 3: polarization at high *T*, readout at low *T*; Sequence 4: polarization at low *T*, readout at low *T*. The MW pulse was always applied during the heating pulse (when the temperature was the highest). (**B**) ODMR spectra measured with the pulse sequences illustrated in **A**. The same integration time (25 ms for each MW frequency) was used for the 4 spectra, and the difference in counts was caused both by different emission efficiencies at different temperatures (1 vs 3 and 2 vs 4) and by different spin polarization rates at different temperatures (1 vs 2 and 3 vs 4).

Supplementary Note 6 | Calibration of temperature at zero-field

The temperature below 700 K was calibrated with the D-T relation from literature (1) , $D(T) = a_0 + a_1 T + a_2 T^2 + a_3 T^3$ with $a_0 = (2.8697 \pm 0.0009)$ MHz, $a_1 = (9.7 \pm 0.6) \times 10^{-2}$ GHz K^{-1} ,$a_2 = (-3.7 \pm 0.1) \times 10^{-4}$ MHz K^{-2}, and $a_3 = (1.7 \pm 0.1) \times 10^{-7}$ MHz K^{-3}.

For temperature higher than 700 K, we measured the ZFS D at different waiting times t_w (see Supplementary Figure 7 for the pulse sequence) and then used extrapolation to determine the temperature right after the heating pulse with the assumption of exponential cooling (verified in Fig. 6 of the main text and Supplementary Figure 5). The ODMR spectra at different t_w were measured in a cycling manner (sequence in Supplementary Figure 7), so as to minimize possible effects due to aging of carbon films (which would reduce the heating efficiency), drifting of NIR laser focus spot, and laser power fluctuations. The protocol is as follows.

{

[P/R→H→wait(t_w=0)→ω_1→P/R→H→wait(t_w=t_1)→ω_1→… P/R→H→wait(t_w=t_n)→ω_1→P/R] x M,

[P/R→H→wait(t_w=0)→ω_2→P/R→H→wait(t_w=t_1)→ω_2→… P/R→H→wait(t_w=t_n)→ω_2→P/R] x M,

… …

[P/R→H→wait(t_w=0)→ω_K→P/R→H→wait(t_w=t_1)→ω_K→… P/R→H→wait(t_w=t_n)→ω_K→P/R] x M,

} x N

P/R – 532 nm laser pulse for spin polarization and readout

H – heating laser pulse (power fixed in the whole unit {…} x N),

wait(t_w=t_i) – a waiting period of t_i (i =1,2, …, n) between the heating pulse and the MW pulse

ω_k – MW pulse with frequency ω_k (fixed within each unit of […] x M)

M – repetition times of the unit […]

N – repetition times of the unit {…}.

We chose n = 5 and t_w = {-0.2, 0.3, 0.6, 1.0, 1.4, 2.2} μs (excluding the NIR AOM delay) for experiments of lower or close to 700 K. For experiments with much higher temperatures, a longer delay was used, e.g., n = 5 and t_w = {-0.2, 0.6, 1.0, 1.4, 2.2 2.6} μs (excluding the NIR AOM delay). Each unit of measurement for the 6 t_w's lasted for about 100 μs. For each MW frequency the measurement lasted for about 20 ms (i.e., M = 200).

We have verified the method by comparing the temperature obtained by extrapolation with that determined by the D-T relation in Supplementary Reference (1) for T < 700 K (see Fig. 2C of the main text). The examples of temperature calibration are shown in Fig. 6 of the main text and the results are summarized in Supplementary Table 1. All the cooling curves are well fit with exponential decay functions with nearly the same cooling time except for the case of OD02. The differences between the cooling rate for OD02 and the others might be caused by the graphitization of the amorphous carbon film at high temperature. The highest temperature recorded was 1004 ± 24 K.

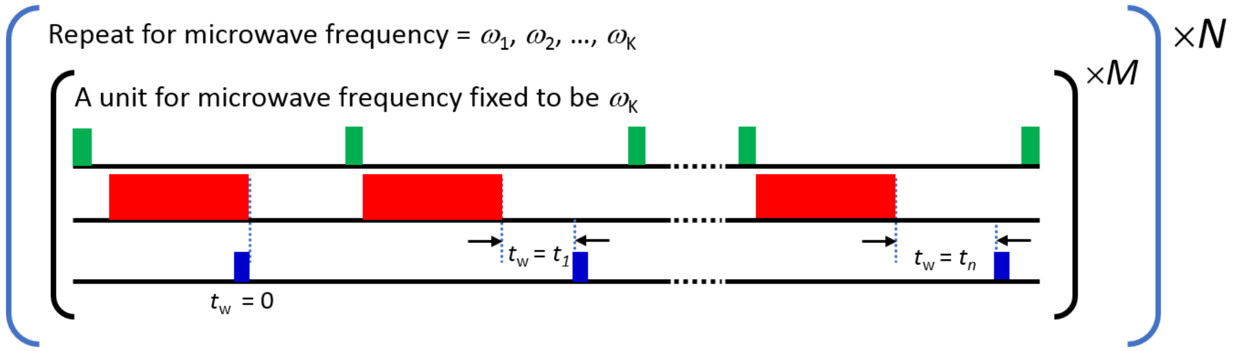

Supplementary Figure 7 | Pulse sequence for temperature calibration. For each MW frequency, the following sequence was cycled *M* times: First, the ODMR of the highest temperature was measured (MW pulse applied right at the end of the NIR heating pulse, $t_w = 0$); then ODMR were measured with the MW pulse applied with certain delay intervals ($t_w = t_1, t_2, \ldots, t_n$). The total time to run the sequence for $n = 5$ was about 100 μs, which was much shorter than the frequency sweep time of the microwave source (20 ms was used for each frequency).

Supplementary Table 1 | Temperature calibration for an ND (NDa2) corresponding to ODMR data in Fig. 6 of the main text. For each NIR laser power (controlled by the OD-number), the zero-field splitting *D* measured right at the end of the heating pulse (t_w=-0.2 μs) was converted to temperature $T_{0,\,DT}$ with the *D*-*T* relation in Supplementary Reference (*1*) if it is < 700 K. The temperature right at the end of the heating pulse obtained by extrapolation of the exponential cooling curve in Fig. 6B of the main text is denoted as $T_{0,\,E}$. The temperatures obtained by the two methods agree well with each other.

	**OD11**	**OD08**	**OD05**	**OD03**	**OD02**
**Cooling time (μs)**	1.2 ± 0.1	1.3 ± 0.1	1.47 ± 0.03	1.3 ± 0.01	0.79 ± 0.02
***D* (MHz)**	2859.1 ± 0.4	2842.0 ± 0.4	2824.9 ± 0.3	2801.5 ± 0.6	2758 ± 1
**$T_{0,\,DT}$ (K)**	409 ± 5	533 ± 5	639 ± 4	N.A.	N.A.
**$T_{0,\,E}$ (K)**	409 ± 18	536 ± 26	648 ± 15	771 ± 4	1004 ± 24

Supplementary Note 7 | Spin coherence at room temperature and raw data of T_1 measurement

The spin coherence of ensemble NV centers in NDs at room temperature is shown in Supplementary Figure 8. An external magnetic field was applied to lift the degeneracy of the 4 crystallographic NV orientations. Typical spin relaxation time (T_1) was a few hundreds of microseconds. The spin coherence time, T_2 =0.91 ± 0.05 μs measured by spin echo and T_2^* = 65 ± 2 ns measured by free-induction decay (FID), were much shorter than those of ensemble NV centers in high-purity bulk diamond (*3*).

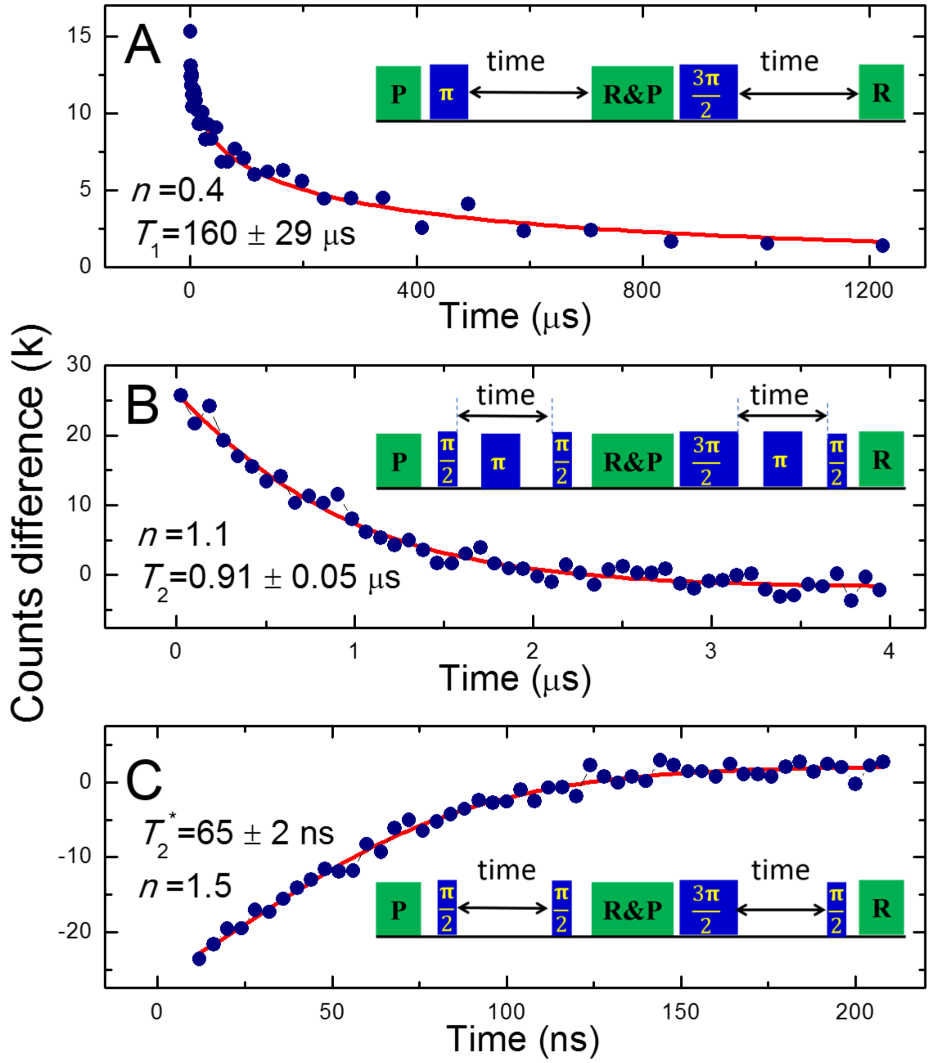

Supplementary Figure 8 | Room temperature spin coherence of ensemble NV centers in an ND. (**A**) Spin relaxation (**B**) Spin echo and (**C**) Free-induction decay (FID) signals of the ensemble NV centers. An external magnetic field of about 80 Gauss was applied to lift the degeneracy of the NV centers of 4 different crystallographic orientations. Insets: pulse sequences for each measurement. To eliminate count fluctuations that are not related to the spin process (e.g. laser power fluctuation and charge dynamics), the difference between the photon counts for the m_s=0 and m_s =-1initial states was taken as the signals.

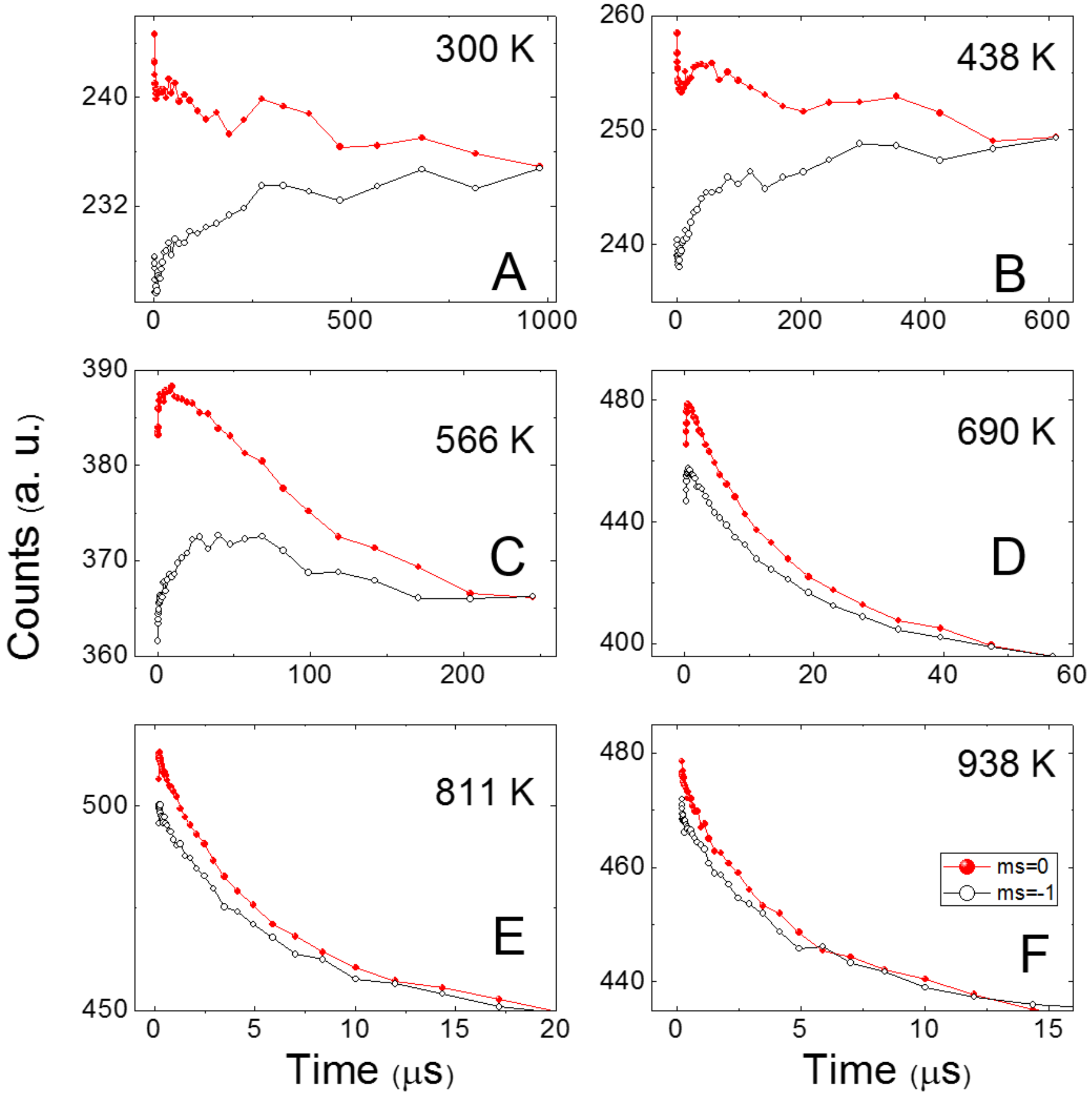

Supplementary Figure 9 | Raw data of T_1 measurement. (**A-F**) Raw data of spin relaxation measurement at different temperatures. Pulse sequence in Fig. 3A of the main text is used. After spin state preparation (m_s=0 and m_s =-1), the NIR pulse heats the ND to a high temperature. The NIR beam maintains this high temperature (the saturated temperature) during the whole spin relaxation process. A 3-μs cooling interval is inserted before the final readout. To eliminate the count fluctuations that are not related to the spin process (e.g. laser power fluctuation and charge dynamics), the difference between the photon counts for the m_s=0 and m_s =-1initial states was taken as the signals. Temperature calibration method is presented in Supplementary Note 8.

Supplementary Note 8 | Temperature calibration in spin coherence measurement

To estimate the ND temperature in spin coherence measurements (data shown in Fig. 3B and 4C of the main text), we measured the full ODMR spectrum (Supplementary Figure 10A) under the same NIR laser power and duty ratio. Supplementary Figure 10B shows the resonant frequencies (peak 1 and peak 4 in Supplementary Figure 10A) of NV centers in one orientation. The zero-field splitting *D* was calculated by averaging the two resonant peaks (the effect of the transverse field (which was less than 10 Gauss), if any, would be much weaker than the variation of *D*). And the calibrated *D*-*T* relation (Fig. 2C of the main text) was used to estimate the ND temperature.

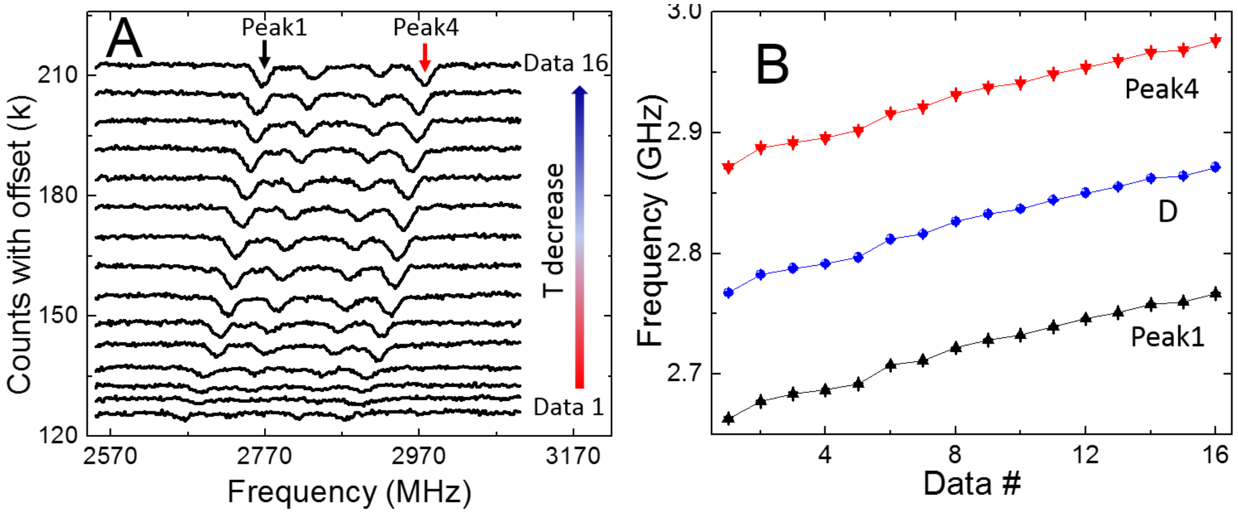

Supplementary Figure 10 | HiT ODMR spectra for coherence measurement. (**A**) ODMR spectra of NDa2 at different temperature. An external magnetic field of 38 Gauss was applied. The resonance peak 1 was used in Rabi oscillation and spin relaxation measurements (data in Fig. 3B and 4C of the main text). The ND temperature was calibrated with the 1st and the 4th resonances, which were associated with the NV centers with the smallest angle (among the 4 crystallographic orientations) from the external magnetic field. (**B**) The frequencies of peak 1 and 4 in **A** and their average (i.e., *D*) for Data # from 1 to 16 (corresponding to curves from bottom to top in **A**). Error bars corresponding to the standard fitting errors.

Supplementary Note 9 | Measurement of magnetization of single Ni NPs.

The measurement protocol is as follows.

For each cooling process (temperature $T_{\mathrm{I}} > T_{\mathrm{II}} > \ldots > T_M$):
{[P/R → T_{I} → ω_1] x M' → [P/R → T_{I} → ω_2] x M' → … → [P/R → T_{I} → ω_K] x M'} x N,
{[P/R → T_{II} → ω_1] x M' → [P/R → T_{II} → ω_2] x M' → … → [P/R → T_{II} → ω_K] x M'} x N,
… …
{[P/R → T_{M} → ω_1] x M' → [P/R → T_{M} → ω_2] x M' → … → [P/R → T_{M} → ω_K] x M'} x N
P/R – 532-nm laser pulse for spin polarization and readout
T_m (m = I, II, …, M) – temperature when the MW pulse was applied (controlled by the waiting duration $t_{\mathrm{w}} = t_m$ after an NIR laser pulse). T_m is fixed in each unit of {…} x N
ω_k (k = 1, 2, …, K) – MW pulse with frequency ω_k
M' – repetition times of each unit […]

The magnetic field from the Ni NP induced splitting and broadening (due to the field gradient within the ND) (see Fig. 5A of main text). We used the splitting of the ODMR spectra to indicate the magnetic field from the nearby Ni NP. As summarized in Supplementary Figure 12, the Ni NP was demagnetized at temperature higher than 615 ±4 K, which agrees well with the Curie temperature T_{C} of bulk nickel, 627 K.

The spontaneous magnetization of this single Ni NP was tracked for 10 cooling processes (Round 1 to 10 in Supplementary Figure 12, and Round 1 to 4 also shown in Fig. 5B of main text). The magnetization was nearly the same if the Ni NP was kept at temperature lower than T_{C}, and new magnetic states could spontaneously emerge if the Ni NP had been heated to a temperature higher than T_{C} (completely demagnetized, T > 615 K). Round 7 was measured with the NIR laser turned off and the ODMR spectra recorded repetitively, in order to estimate the statistic error of this experiment. The difference in ODMR splitting (e.g., that between Round 3 and 4) was much larger than the statistic error.

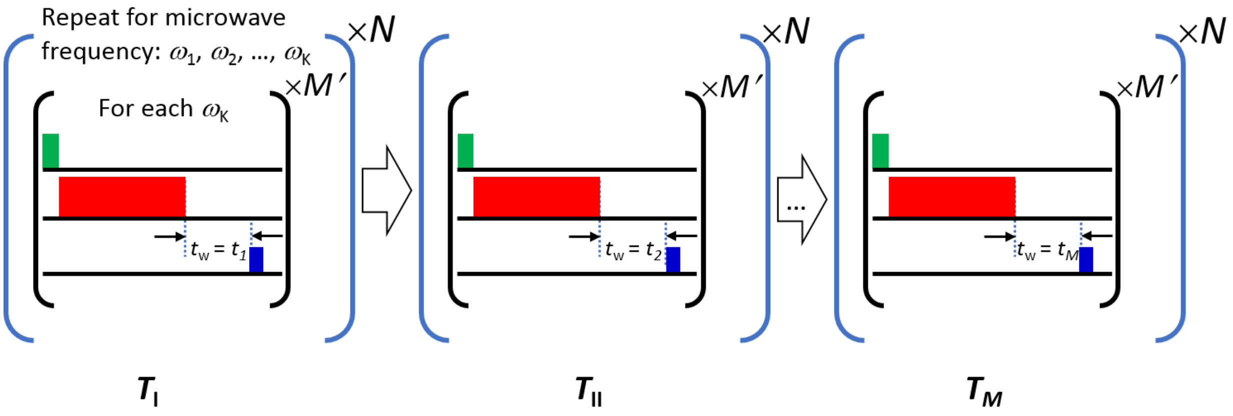

Supplementary Figure 11 | Pulse sequence for the Ni NP experiments. The magnetization of a Ni NP was measured in a round of cooling process. The highest temperature was set by the power of the NIR laser pulse (saturated heating). The different temperatures ($T_{\mathrm{I}} > T_{\mathrm{II}} > \ldots > T_M$) were controlled by the waiting duration t_{w}. For each temperature T_{m}, the zero-field ODMR spectrum was measured in a frequency-cycling manner.

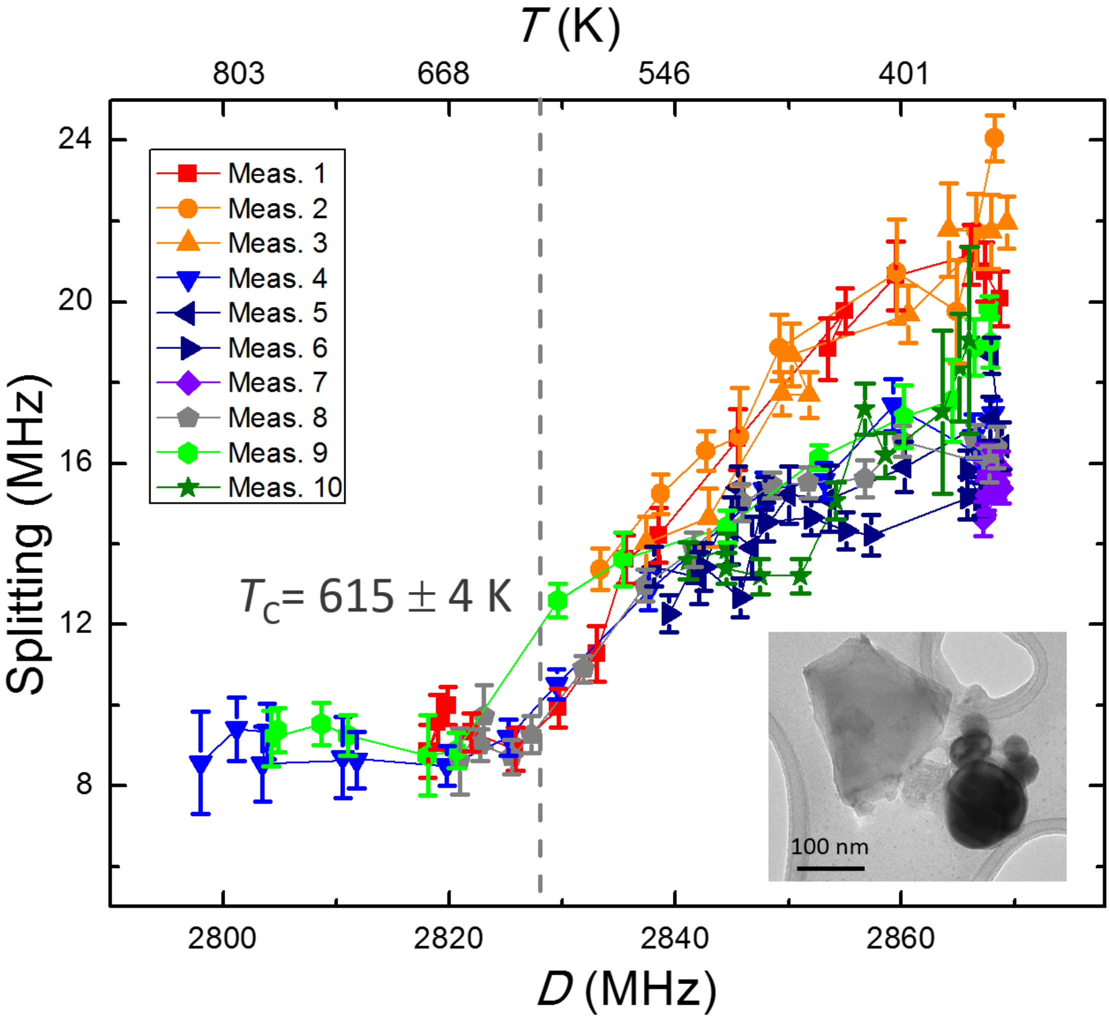

Supplementary Figure 12 | Spontaneous magnetization of a single Ni NP. The ODMR splitting induced by the Ni NP was measured as a function of the ZFS D (corresponding temperature shown in the top axis). The measurement was carried out from round 1 to round 10 (indicated by different symbols), each of which contained a sequence shown in Supplementary Figure 11. Round 1, 4, 8, and 9 were started at a temperature higher than T_c (about 615 K, corresponding to D = 2829 MHz, indicated by the vertical dotted line). Round 7 was repetitive measurement at room temperature, which gives the statistic error of this method. Error bars are fitting error of the ODMR spectra (fitting by a double-peak Lorentzian function, see Fig. 5A of the main text). Inset: TEM image of the Ni NP and the ND on the carbon film that were measured. Error bars in corresponding to the standard fitting errors.

Supplementary Note 10 | Equations for sensitivities

The magnetic field sensitivity ($\eta_{\mathrm{B,cw}}$) of an ND with ensemble NV centers was estimated by the following formula (*4*)

$$\eta_{\mathrm{B,cw}} = \frac{h}{g\mu_B}\frac{\Delta\omega}{C\sqrt{L}}, \tag{3}$$

where L is the photon count rate, h is the Planck constant, $g = 2.0$ is the Lande g-factor, μ_B is the Bohr magneton, and $\Delta\omega$ is the width of the ODMR resonant dip. In the Ni NP experiment, the experimental measured values were: $L = 8$ Mps, $\Delta\omega = 10$ MHz, and $C = 0.05$. And the estimated $\eta_{\mathrm{B,cw}} = 2.5\ \mu\mathrm{T\ Hz}^{-1/2}$.

The temperature sensitivity ($\eta_{\mathrm{T,cw}}$) of a ND nanothermometer was estimated by the following formula (*2*)

$$\eta_{\mathrm{T,cw}} = \frac{\Delta\omega}{C\sqrt{L}|dD/dT|}. \tag{4}$$

For $\mathrm{d}D/\mathrm{d}T = 240$ kHz K^{-1} at temperature close to 1000 K, the sensitivity $\eta_{\mathrm{T,cw}} = 250$ mK $\mathrm{Hz}^{-1/2}$.

Supplementary References

1. D. M. Toyli *et al.*, Measurement and control of single nitrogen-vacancy center spins above 600 K. *Phys. Rev. X.* **2,** 031001 (2012). DOI: https://doi.org/10.1103/PhysRevX.2.031001
2. N. Wang *et al.*, Magnetic Criticality Enhanced Hybrid Nanodiamond Thermometer under Ambient Conditions. *Phys. Rev. X.* **8**, 011042 (2018). DOI: https://doi.org/10.1103/PhysRevX.8.011042
3. P. L. Stanwix *et al.*, Coherence of nitrogen-vacancy electronic spin ensembles in diamond. *Phys. Rev. B.* **82**, 201201 (2010).
4. L. Rondin *et al.*, Magnetometry with nitrogen-vacancy defects in diamond. *Rep. Prog. Phys.* **77,** 056503 (2014).
